# Supplementary material for: The role of non-uniform magnetization texture for magnon–magnon coupling in an antidot lattice
Source: Sci Rep. 2024 May 20;14:11501. doi: 10.1038/s41598-024-61246-5 (PMC11106278; doi:10.1038/s41598-024-61246-5)
Supplement: Supplementary file 1 — Supplementary Figures. [file 41598_2024_61246_MOESM1_ESM.pdf]

Supplemental material
The role of non-uniform magnetization texture for magnon–magnon coupling in an antidot lattice

Mathieu Moalic, Mateusz Zelent, Krzysztof Szulc, and Maciej Krawczyk
Institute of Spintronics and Quantum Information,
Faculty of Physics, Adam Mickiewicz University, Poznan, Poland
(Dated: January 19, 2024)

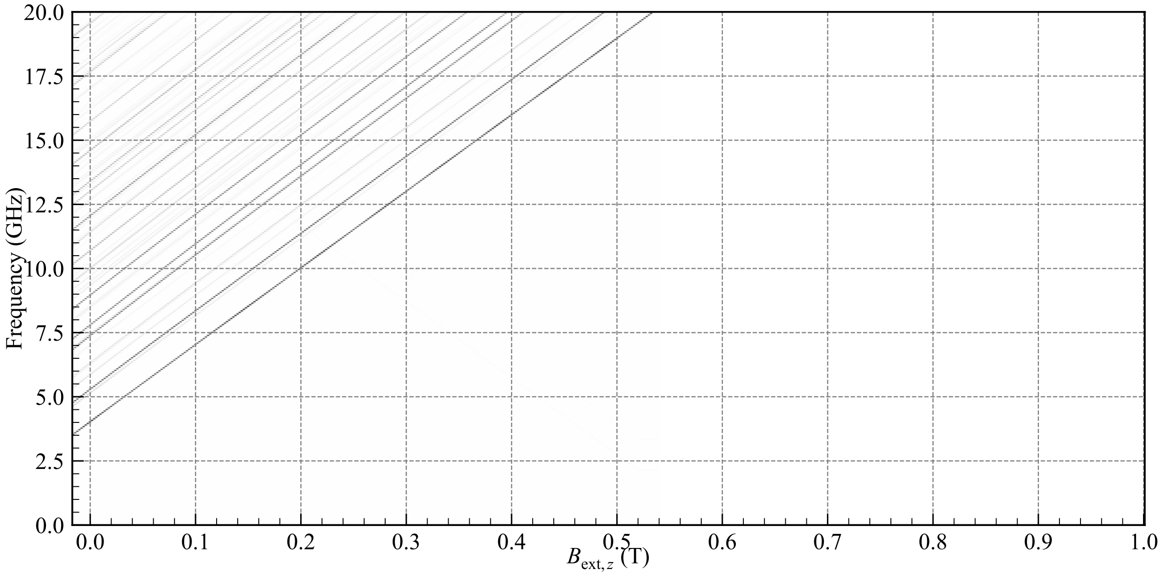

FIG. S1. Evolution of the spin waves (SWs) resonance spectra for the antidot lattice in dependence on the static external out-of-plane magnetic field. The line intensity corresponds to the SW amplitude. This is depicted in green in Fig. 2 of the main text.

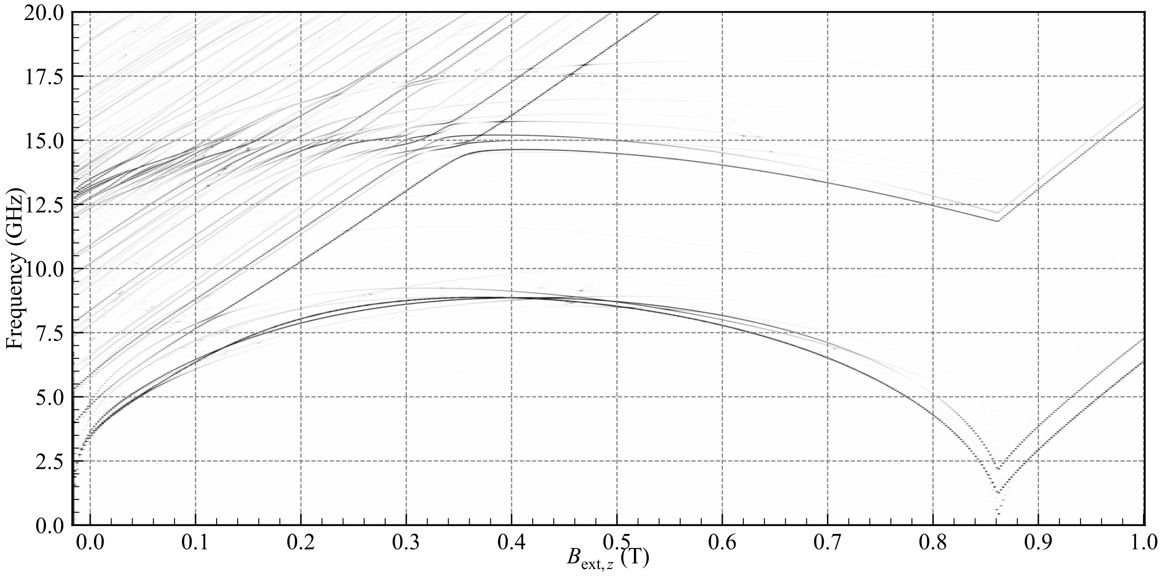

FIG. S2. Evolution of the SW resonance spectra for the antidot lattice with modified rim in dependence on the static external out-of-plane magnetic field. The line intensity corresponds to the SW amplitude. This is depicted in red in Fig. 2, 3 and 4 of the main text.

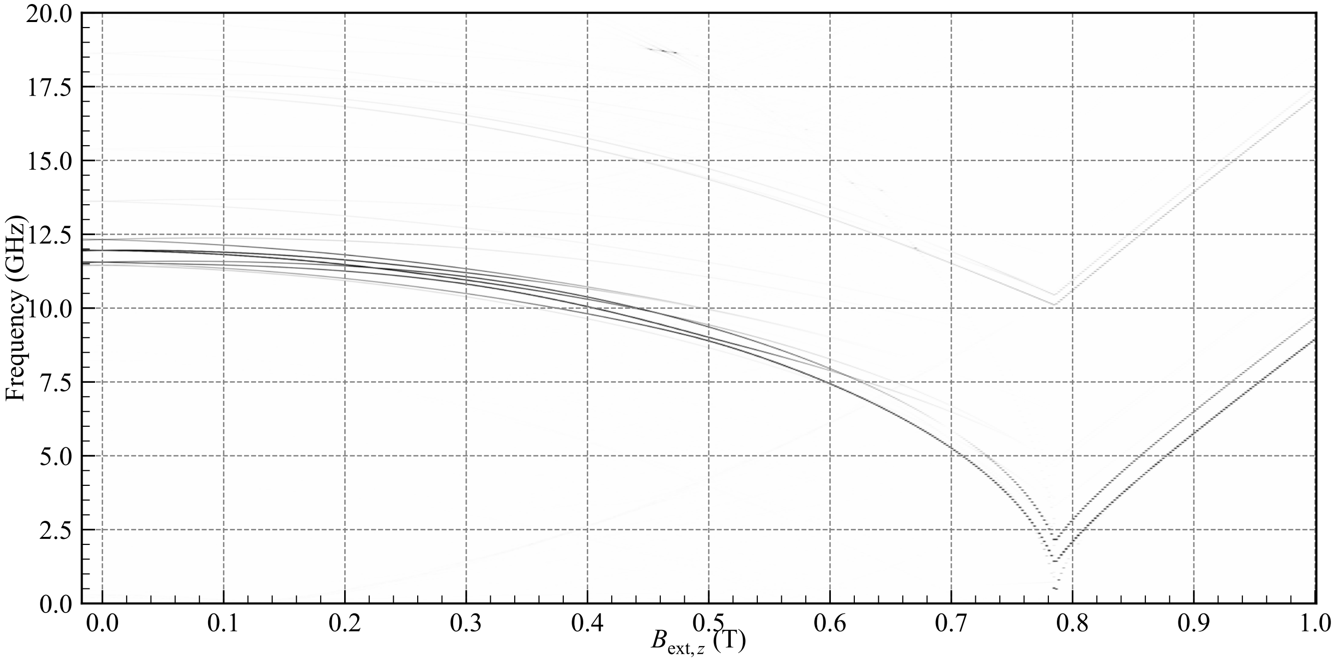

FIG. S3. Evolution of the SW resonance spectra for the rings lattice in dependence on the external out-of-plane magnetic field. The line intensity corresponds to the SW amplitude. This is depicted in purple in Fig. 2 and 4 of the main text.

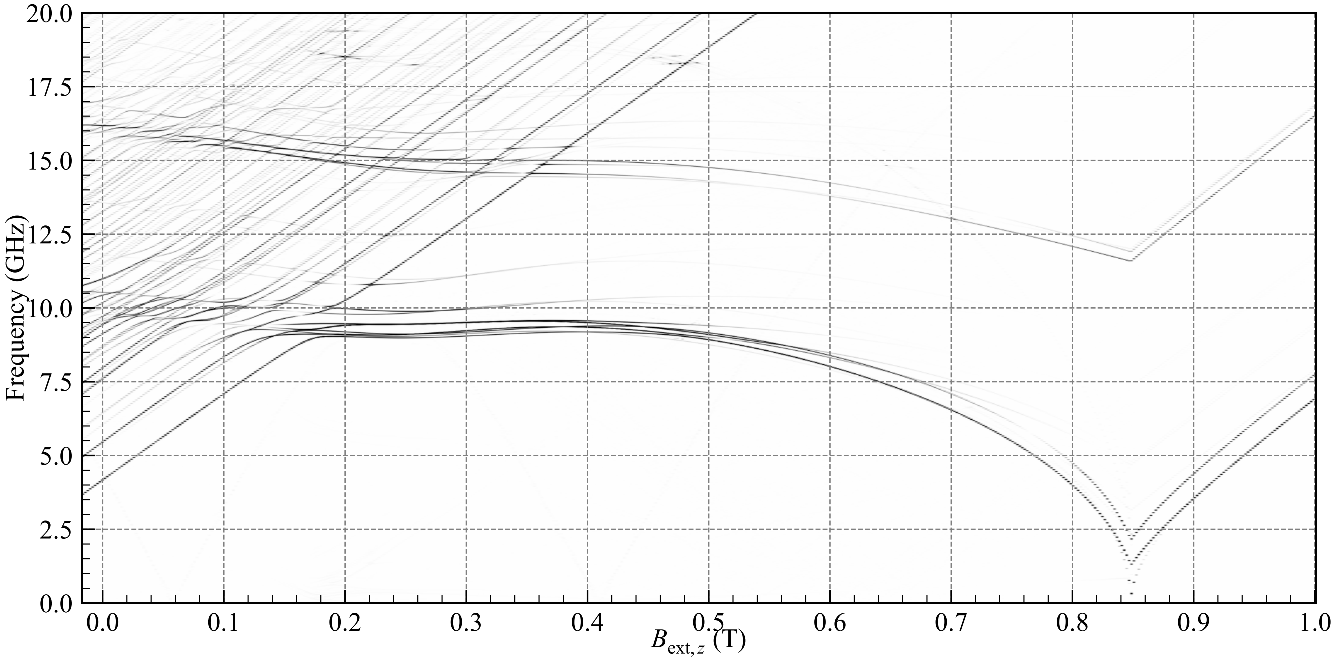

FIG. S4. Evolution of the SW resonance spectra for the antidot lattice with modified rim with the separation between bulk and rim in dependence on the static external out-of-plane magnetic field. The line intensity corresponds to the SW amplitude. This is depicted in blue in Fig. 4 of the main text.

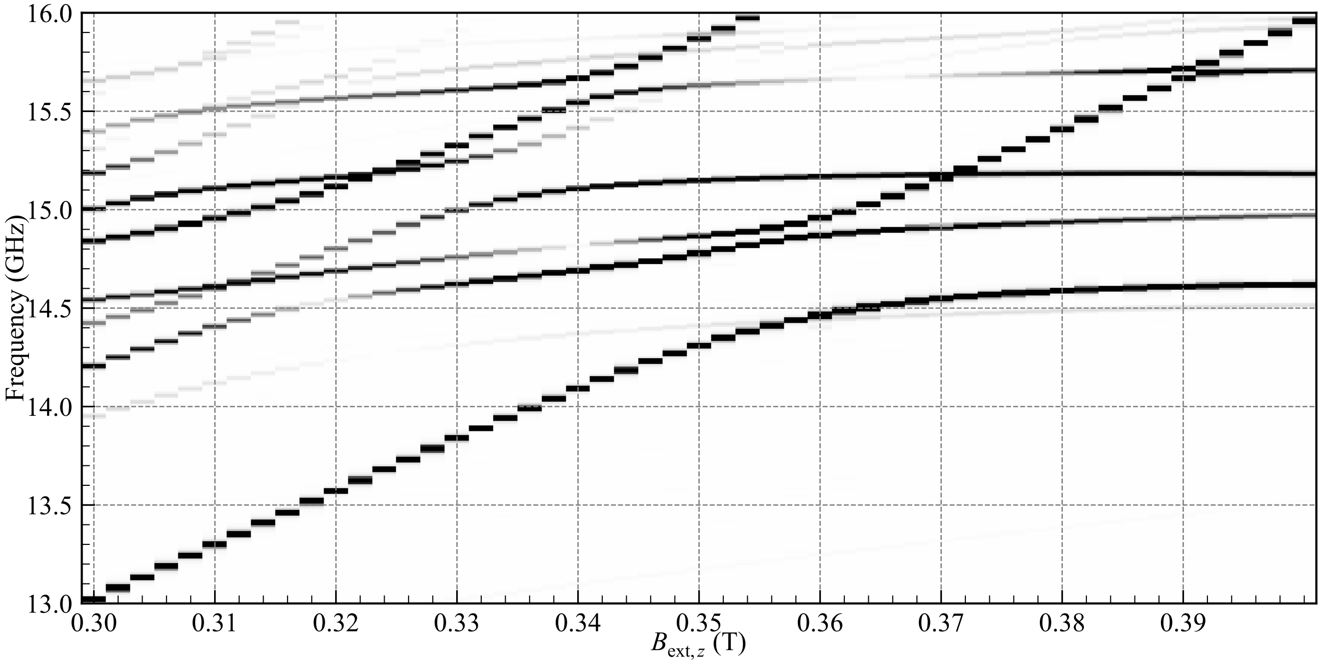

FIG. S5. A zoom-in on Fig. S2 which represents the evolution of the SW resonance spectra for the antidot lattice with modified rim in dependence on the static external out-of-plane magnetic field. The line intensity corresponds to the SW amplitude. This is depicted in red in Fig. 5 of the main text.

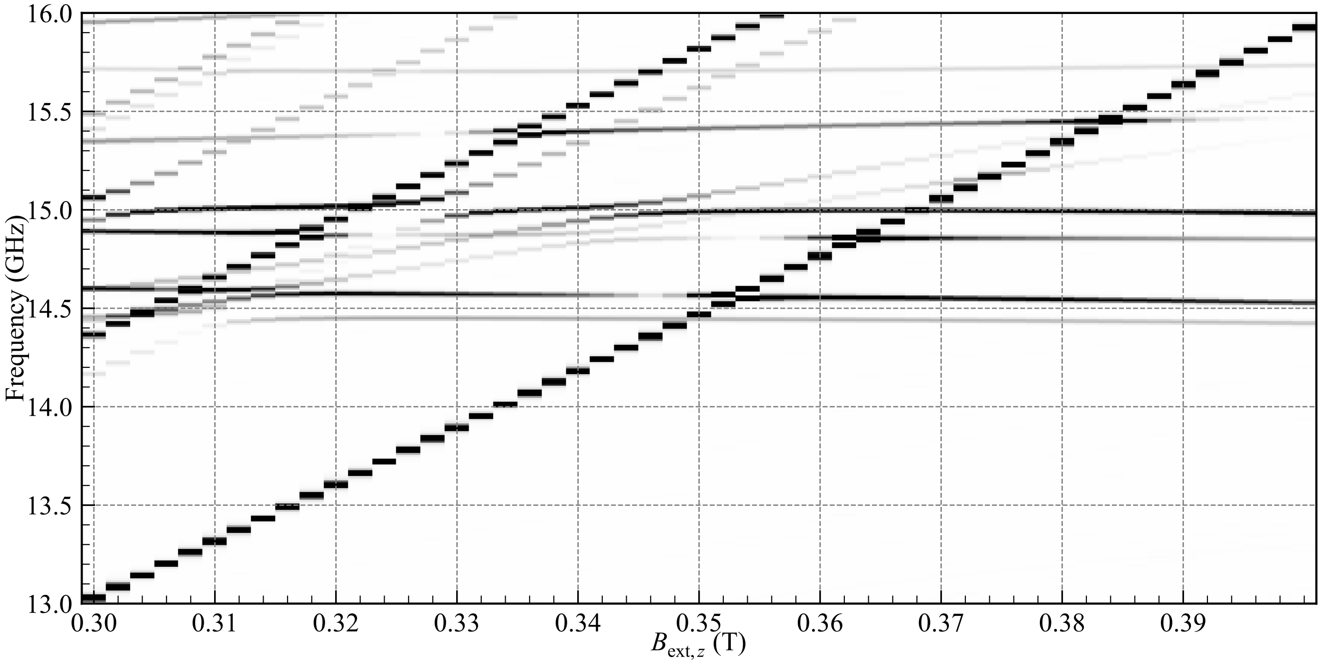

FIG. S6. A zoom-in on Fig. S4 which represents the evolution of the SW resonance spectra for the antidot lattice with modified rim with the separation between bulk and rim in dependence on the external out-of-plane magnetic field. The line intensity corresponds to the SW amplitude. This is depicted in blue in Fig. 5 of the main text.

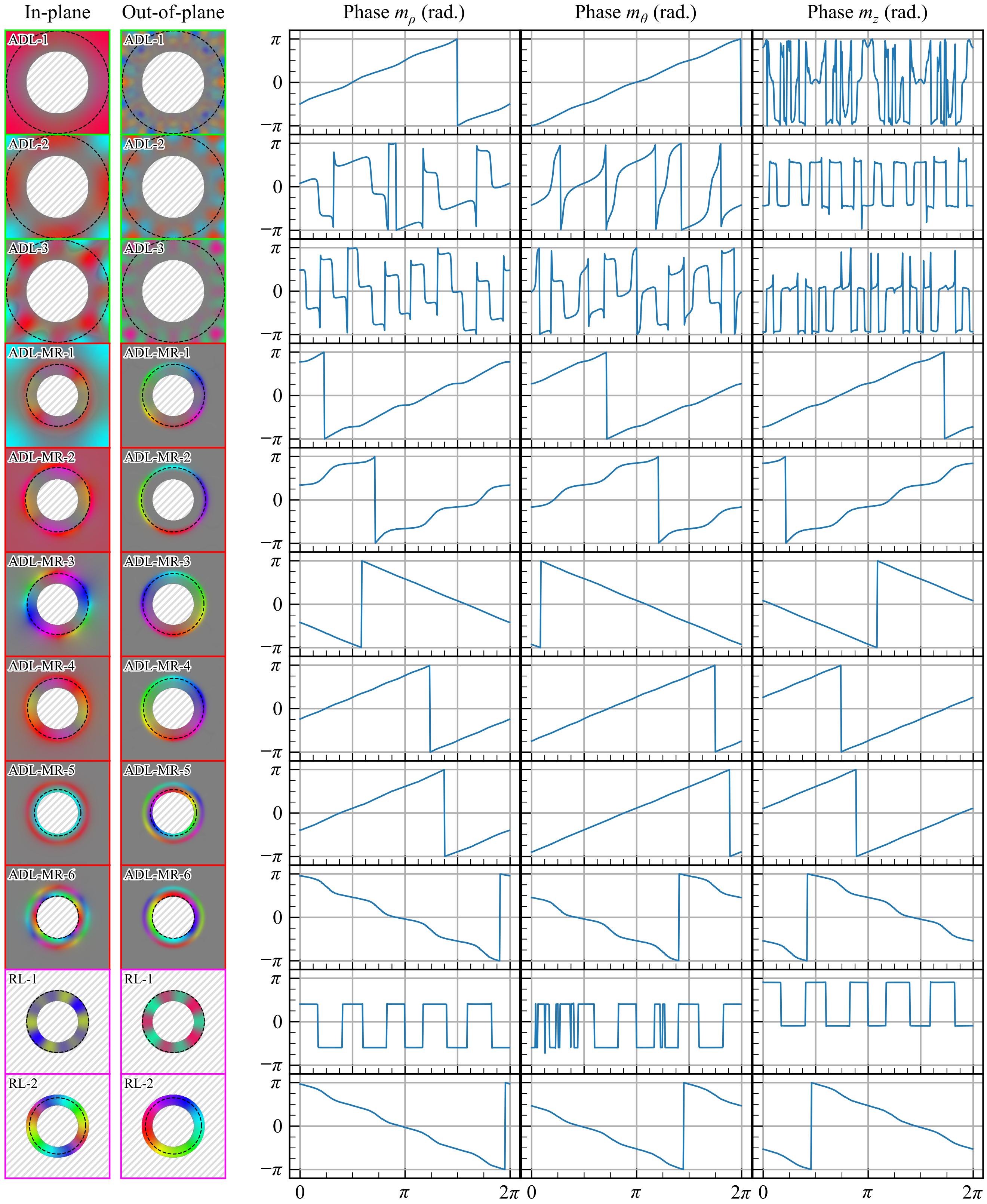

FIG. S7. Detailed views of each mode from Fig. 2 in the main text are presented. The first and second columns represent the in-plane and out-of-plane dynamic components of the magnetization for the selected modes, respectively. The hue indicates the phase of the in-plane dynamic magnetization, while the saturation reflects its spatial amplitude. The dotted line highlights the location of the peak amplitude of each selected mode. The third, fourth, and fifth columns display the cross-sections of the phase along the dotted line, which circles the rim of the mode, for the m_ρ, m_θ and m_z components, respectively. The azimuthal order of the modes and their chirality are deduced from the phase of the m_z component.

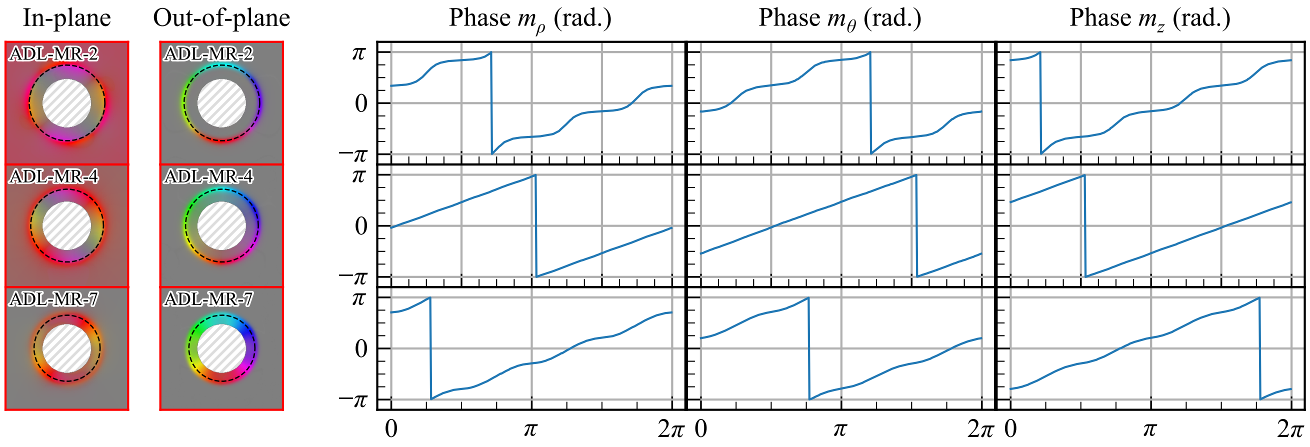

FIG. S8. Detailed views of each mode from Fig. 3 in the main text are presented. The first and second columns represent the in-plane and out-of-plane dynamic components of the magnetization for the selected modes, respectively. The hue indicates the phase of the in-plane dynamic magnetization, while the saturation reflects its spatial amplitude. The dotted line highlights the location of the peak amplitude of each selected mode. The third, fourth, and fifth columns display the cross-sections of the phase along the dotted line, which circles the rim of the mode, for the m_ρ, m_θ and m_z components, respectively. The azimuthal order of the modes and their chirality are deduced from the phase of the m_z component.

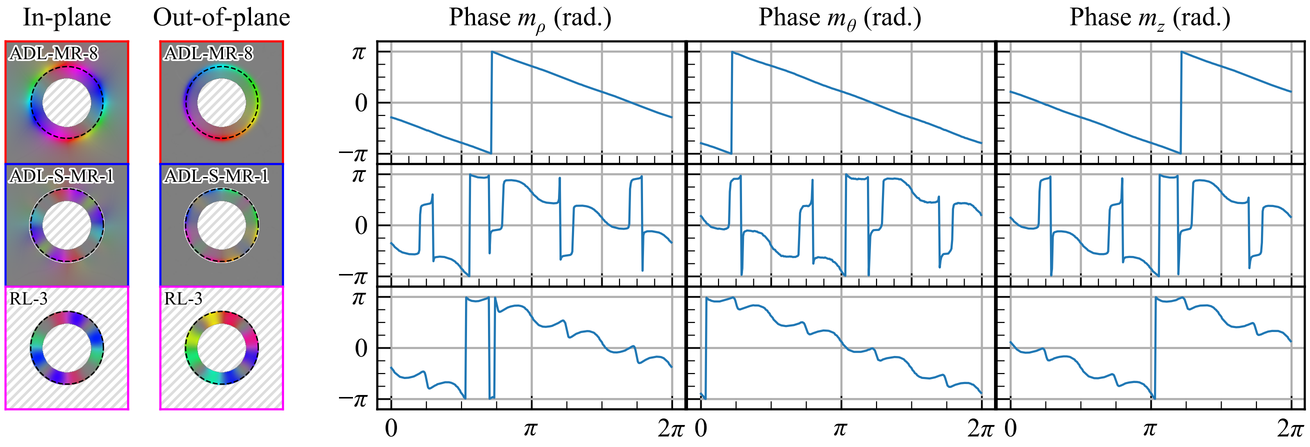

FIG. S9. Detailed views of each mode from Fig. 4 in the main text are presented. The first and second columns represent the in-plane and out-of-plane dynamic components of the magnetization for the selected modes, respectively. The hue indicates the phase of the in-plane dynamic magnetization, while the saturation reflects its spatial amplitude. The dotted line highlights the location of the peak amplitude of each selected mode. The third, fourth, and fifth columns display the cross-sections of the phase along the dotted line, which circles the rim of the mode, for the m_ρ, m_θ and m_z components, respectively. The azimuthal order of the modes and their chirality are deduced from the phase of the m_z component.

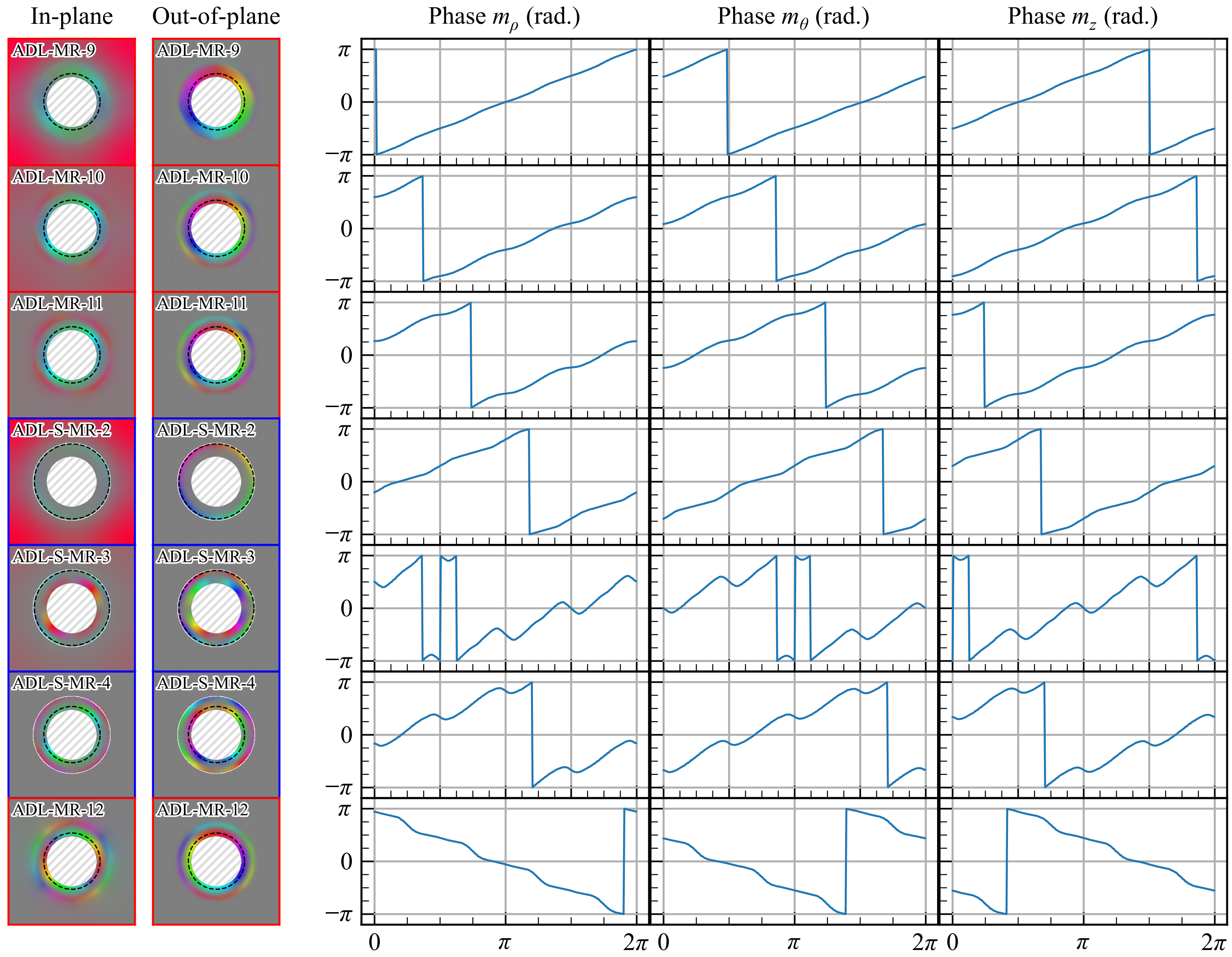

FIG. S10. Detailed views of each mode from Fig. 5 in the main text are presented. The first and second columns represent the in-plane and out-of-plane dynamic components of the magnetization for the selected modes, respectively. The hue indicates the phase of the in-plane dynamic magnetization, while the saturation reflects its spatial amplitude. The dotted line highlights the location of the peak amplitude of each selected mode. The third, fourth, and fifth columns display the cross-sections of the phase along the dotted line, which circles the rim of the mode, for the m_ρ, m_θ and m_z components, respectively. The azimuthal order of the modes and their chirality are deduced from the phase of the m_z component.
